# Investigating the geometrical preferences of a flexible benzimidazolone-based linker in the synthesis of coordination polymers

**DOI:** 10.1098/rsos.171064

**Published:** 2017-12-06

**Authors:** Corey L. Jones, Elizabeth A. Marsden, Adam C. Nevin, Benson M. Kariuki, Mohan M. Bhadbhade, Adam D. Martin, Timothy L. Easun

**Affiliations:** 1 School of Chemistry, Cardiff University, Main Building, Park Place, Cardiff CF10 3AT, UK; 2 School of Chemistry, Mark Wainwright Analytical Centre, The University of New South Wales, Sydney, NSW 2052, Australia; 3 School of Chemistry, The Australian Centre for Nanomedicine and the ARC Centre of Excellence in Convergent Bio-Nano Science, The University of New South Wales, Sydney, NSW 2052, Australia

**Keywords:** coordination polymer, coordination network, metal–organic framework, crystallization, flexible linker

## Abstract

A series of new group 2 coordination polymers, **MgL** ={MgL(H_2_O)(DMF)_0.75_}_∞_, **CaL** = {CaL(DMF)_2_}_∞_, **SrL** = {SrL(H_2_O)_0.5_}_∞_ and **BaL** = {BaL(H_2_O)_0.5_}_∞_, were synthesized using a flexible benzimidazolone diacetic acid linker (**H**_2_**L**) in which the two carboxylic acid binding sites are connected to a planar core via {–CH_2_–} spacers that can freely rotate in solution. In a ‘curiosity-led' diversion from group 2 metals, the first row transition metal salts Mn^2+^, Cu^2+^ and Zn^2+^ were also reacted with **L** to yield crystals of **MnL =** {MnL(DMF)(H_2_O)_3.33_}_∞_, **Cu_3_L_2_** = {Cu_3_L_2_(DMF)_2_(CHO_2_)_2_}_∞_ and **ZnL** = {ZnL(DMF)}_∞_. Crystal structures were obtained for all seven materials. All structures form as two-dimensional sheets and contain six-coordinate centres, with the exception of **ZnL,** which displays tetrahedrally coordinated metal centres, and **Cu_3_L_2_**, which contains square planar coordinated metal centres and Cu paddle-wheels. In each structure, the linker adopts one of two distinct conformations, with the carboxylate groups either *cis* or *trans* with respect to the planar core. All materials were also characterized by powder X-ray diffraction and thermogravimetric analysis.

## 1. Introduction

Self-assembly processes frequently occur in biology, with detrimental examples including formation of amyloid-β plaques within the brain that may lead to Alzheimer's disease [1,2]. The ability of small molecules to bind to or interfere with these protein assemblies is well documented, with molecules such as Thioflavin T now used as a diagnostic tool for amyloid formation [3–6]. Benzimidazolone represents a somewhat underused heterocyclic scaffold in this regard. Its ability to mimic the amino acid tryptophan has led to synthetic analogues finding uses as non-nucleoside reverse transcriptase inhibitors for HIV-1, histamine H_3_-receptor antagonists and HSP-90 inhibitors [7–9]. The benzimidazolone moiety has also been appended to the N-terminus of flexible short peptides, resulting in self-assembly of these peptides into tunable hydrogels [10].

In the field of coordination polymers, in which Robson and Hoskins published pioneering work almost 30 years ago [11,12], innumerable structures have been reported that consist of metal nodes connected by *rigid* organic linkers. Flexible metal–organic frameworks (MOFs) have been reported since the early 2000s, with examples including a nanoporous interpenetrated iron framework by Halder *et al.* [13] in which substantial flexibility with guest uptake and release was observed, and a zinc framework by Dybtsev *et al.* [14] that demonstrated sufficient flexibility to show unusual guest-dependent dynamic behaviour. Several notable reviews have been published over the last few years, describing various frameworks that demonstrate flexibility upon response to stimuli [15–17]. Interest is also growing in the use of more flexible linkers and how they affect particularly the assembly of MOFs, with several important examples being reported by Rosseinsky and co-workers. In 2010, they synthesized a framework with a peptide linker, which led to changes in the pore conformation [18], and in one of the most recent examples in the field, they have reported the synthesis of two indium frameworks, In(OH)CSA and In(OH)PDG, with flexible amide functionalized linkers *N*-(4-carboxyphenyl)succinamic acid (CSA) and *N,N'*-(1,4-phenylenedicarbonyl)diglycine (PDG) [19]. These linkers were described as flexible due to the presence of an sp^3^ carbon in the backbone of each molecule. The frameworks formed with both linkers contain indium hydroxide chains of corner-sharing {InO_4_(OH)_2_} octahedra which are interconnected by the dicarboxylate linkers to form stacked two-dimensional (2D) layers. However, the supramolecular interactions between linkers are different in the two frameworks as a result of differing conformational configurations of the two linkers, leading to differently shaped pores and orientations of functional groups. Benzimidazolone diacetic acid can be classed as a flexible molecule by the same definition, albeit one with a slightly more limited conformational flexibility, and is therefore of structural interest both for the self-assembled coordination polymers that it can form and for its structural preferences in biological systems.

There are numerous reports of benzimidazolone-based derivatives published in the literature. One of the first examples was described in 1975 whereby halogenated benzimidazolone substitutes were reported as fire-retardant monomers or further halogenated for use as fireproofing agents [20]. Within the last 20 years, benzimidazolone analogues have gained notable attention in biological research. In 2000, Dannhardt & Kohl [21] reported the synthesis of a series of differently substituted benzimidazole derivatives, for which their ability to displace [^3^H]MDL-105,519 ([(*E*)-3-(2-phenyl-2-carboxyethenyl)-4,6-dichloro-1H-indole-2-carboxylic acid]) in rat cortical membranes was explored. In 2009, Zawahir *et al.* [22] presented the synthesis of small-molecule APE1 (human apurinic/apyrimidinic endonuclease 1) inhibitors, including several containing benzimidazolone structures. They found that all the potent molecules showed an inhibitory activity of below 10 µM and that they were selective for APE1 inhibition. More recently, substituted benzimidazolones have been reported as antiviral agents, where examples include compounds that have been found to be inhibitors of HIV [23,24]. Benzimidazolone derivatives have also been found to have antifungal and antibacterial properties [25,26].

In this study, the synthesis of seven coordination polymers and the resulting differences in structure are discussed. Group 2 metals were chosen as they do not display strong geometrical preferences, thereby maximizing the possibility of forming a series of coordination polymers. Owing to the increasing size of the metal ions upon descending group 2, an increase in the coordination number around the metal centre was predicted [27]. A ‘curiosity-led' additional study was undertaken into the transition-metal benzimidazolone diacetic acid complexes that form with Mn^2+^, Cu^2+^ and Zn^2+^ to compare the ligand geometries in the resulting coordination polymers. These metals have their own geometrical preferences (depending on the number of d-electrons), which influence the coordination geometry of the subsequent materials [28]. The X-ray crystal structures obtained with manganese, copper and zinc are reported, and are the result of the interplay of metal geometry preferences and the linker structure. All seven coordination polymers synthesized formed 2D networks and the linker was seen to exist in two different conformations—*cis* and *trans.* They also exhibit low porosity (calculated by PLATON SOLV [29]) due primarily to the small linker size. The bulk samples were analysed by thermogravimetric analysis (TGA) and their crystallinity was investigated by powder X-ray diffraction (PXRD).

## 2. Material and methods

The full syntheses and characterization details are reported in the electronic supplementary material.

### 2.1. Materials

Phenylenediamine and sodium hydride were purchased from Sigma-Aldrich. Urea was obtained from AnalaR. Ethyl bromoacetate and anhydrous dimethylformamide (DMF) were purchased from ACROS Organics. All metal nitrate salts were purchased from Alfa Aesar. Ethylene glycol, DMF and common laboratory solvents were purchased from Fisher Chemical. All reagents were used without any further purification.

### 2.2. Characterization

Single crystal X-ray diffraction data for the group 2, manganese and zinc coordination polymers were collected on an Agilent SuperNova Dual Atlas four-circle diffractometer with either a Cu source (**SrL** and **BaL**) or a Mo source (**MgL**, **CaL**, **MnL** and **ZnL**) and CCD detector. For **Cu_3_L_2_**, data were collected on a Bruker Apex II with a Mo source and CCD detector. Data integration and reduction was performed by the CrysAlisPro system software. All structures were solved by direct methods using Olex2 [30], with the ShelXT and ShelXS structure solution program [31,32], refined with the ShelXL refinement package using least-squares minimization [33]. The H atoms on water molecules could not be located, but are included in the formula sums. PXRD was collected at room temperature on an X'Pert PRO PANalytical Chiller 59 diffractometer using CuK*α* radiation. The samples were loaded onto zero-background silicon wafers directly from the reaction solution. TGA was performed using a PerkinElmer Pyris 1 thermogravimetric analyser. The samples were heated from 25°C to 600°C under a flow of air (20 ml min^−1^), using a heating rate of 5°C min^−1^. A SHIMADZU IRAffinitt-1S spectrometer was used to obtain IR data. ^1^H and ^13^C nuclear magnetic resonance spectra were recorded on a Bruker 400 UltraShield^TM^ spectrometer and referenced to the residual solvent peak.

### 2.3. Synthesis of benzimidazolone diacetic acid (**H**_**2**_**L**)

The full experimental details, spectroscopic analysis and purity of the compounds are included in the electronic supplementary material.

### 2.4. Synthesis of **MgL**

Mg(NO_3_)_2_·6H_2_O (31 mg, 0.12 mmol) and **H_2_L** (10 mg, 0.04 mmol) were dissolved in DMF (2 ml) in an 8 ml Wheaton vial. Ethanol (0.5 ml) and water (0.2 ml) were added to the solution which was then sealed and heated at 80°C for 2 days to give colourless crystals. The crystals were used to seed a second reaction, under the same conditions, yielding larger colourless crystals suitable for X-ray single crystal diffraction analysis.

### 2.5. Synthesis of **CaL**

Ca(NO_3_)_2_·4H_2_O (71 mg, 0.30 mmol) and **H_2_L** (25 mg, 0.10 mmol) were dissolved in DMF (2.5 ml) in an 8 ml Wheaton vial. Formic acid (11 µl) was added to the solution, which was then sealed and heated at 100°C for 24 h, yielding colourless crystals.

### 2.6. Synthesis of **SrL**

Sr(NO_3_)_2_ (25 mg, 0.12 mmol) and **H_2_L** (10 mg, 0.04 mmol) were dissolved in DMF (2.5 ml) in an 8 ml Wheaton vial. Then, 0.4 M HCl (1 ml) was added to the solution, which was then sealed and heated at 80°C for 24 h, yielding colourless crystals.

### 2.7. Synthesis of **BaL**

Ba(NO_3_)_2_ (31 mg, 0.12 mmol) and **H_2_L** (10 mg, 0.04 mmol) were dissolved in DMF (2.5 ml) in an 8 ml Wheaton vial. Then, 0.4 M HCl (1 ml) was added to the solution, which was then sealed and heated at 80°C for 24 h, yielding colourless crystals.

### 2.8. Synthesis of **MnL**

Mn(NO_3_)_2_·4H_2_O (30 mg, 0.12 mmol) and **H_2_L** (10 mg, 0.04 mmol) were dissolved in DMF (2.5 ml) in an 8 ml Wheaton vial. Then, 0.2 M HCl (1 ml) was added to the solution, which was then sealed and heated at 80°C for 2 days, yielding colourless crystals.

### 2.9. Synthesis of **Cu**_**3**_**L**_**2**_

Cu(NO_3_)_2_·3H_2_O (73 mg, 0.30 mmol) and **H_2_L** (25 mg, 0.10 mmol) were dissolved in DMF (2.5 ml) in an 8 ml Wheaton vial. Formic acid (11 µl) was added to the solution, which was then sealed and heated at 90°C for 24 h, yielding green crystals.

### 2.10. Synthesis of **ZnL**

Zn(NO_3_)_2_·6H_2_O (90 mg, 0.30 mmol) and **H_2_L** (25 mg, 0.10 mmol) were dissolved in DMF (2.5 ml) in an 8 ml Wheaton vial. Formic acid (11 µl) was added to the solution, which was then sealed and heated at 80°C for 24 h, yielding a colourless product. Single crystals were obtained by using the same conditions and heating the reaction to 100°C for 24 h.

## 3. Results and discussion

### 3.1. Linker and coordination polymer synthesis

**Scheme 1.**
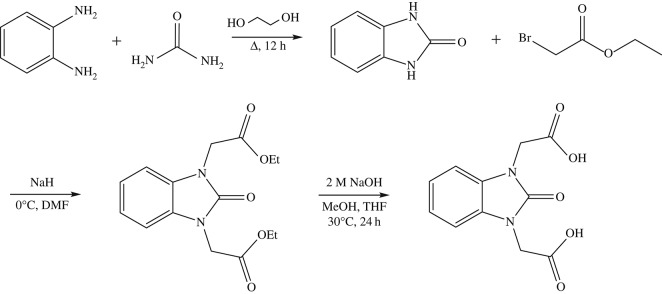
Three-step synthesis of benzimidazolone diacetic acid (**H_2_L**).

The synthesis of **H_2_L** was first reported in 1985 [34]; however, in this study we have used a novel route avoiding the use of solid sodium. The three-step synthesis is highlighted in scheme 1. The first step followed a literature preparation in which phenylenediamine and urea refluxed in ethylene glycol produced benzimidazolone in a 60% yield [35]. The subsequent alkylation reaction with benzimidazolone, sodium hydride and ethyl bromoacetate formed benzimidazolone diethyl acetate ester in a 66% yield. The final step was a hydrolysis reaction in which the ester was stirred overnight at 30°C in NaOH followed by acidification to produce the linker in 56% yield.

**Figure 1.**
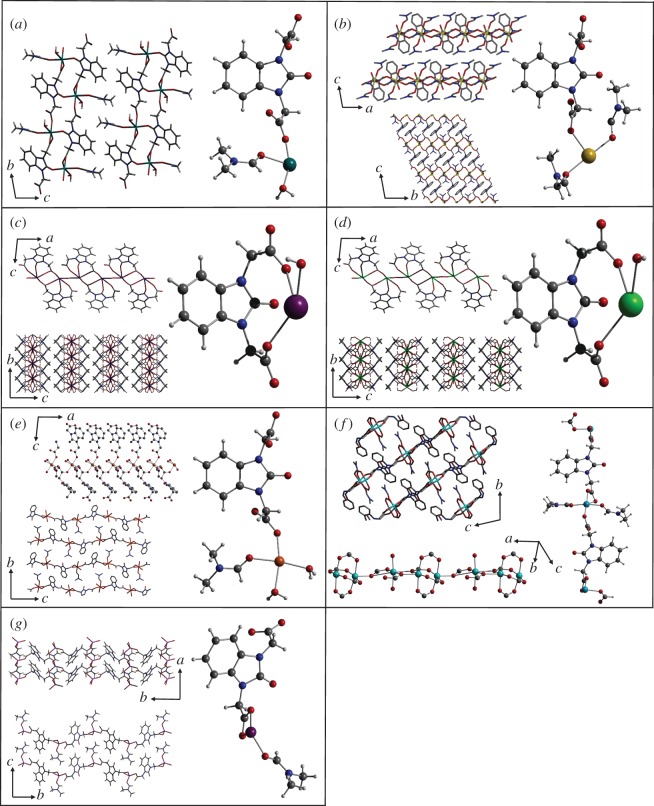
Crystal structures of the seven coordination polymers using the flexible benzimidazolone diacetic acid linker: (*a*) **MgL**, (*b*) **CaL**, (*c*) **SrL**, (*d*) **BaL**, (*e*) **MnL**, (*f*) **Cu_3_L_2_** and (*g*) **ZnL**. For each structure, the asymmetric unit is shown with views along various axes.

The linker was then used in a series of reactions with metal nitrate salts producing coordination polymers. A large range of conditions were attempted in order to achieve single-crystal growth (electronic supplementary material, table S1). The most successful of these are reported in the Material and methods section above, and the crystal structures obtained for the seven coordination polymers are shown in figure 1.

### 3.2. Group 2 metal coordination polymers

**MgL**. Single-crystal X-ray analysis reveals that **MgL** crystallizes in the triclinic space group 1 (electronic supplementary material, table S2). The asymmetric unit contains one Mg^2+^ ion, one **L** molecule, one coordinated water molecule and one coordinated DMF molecule (figure 1*a*). Only one type of magnesium ion is present in the structure, in which each metal centre is octahedrally coordinated to six oxygen atoms. O(1) is from a water molecule, O(2) is from a DMF molecule and O(3) is the ketone oxygen from **L**. O(4), O(5) and O(6) are carboxylate oxygens from three different **L** molecules. The linker bridges between chains of magnesium ions running along the *a*-axis, where it binds with the carboxylate groups in a *trans*-conformation on opposite sides of the plane of the central rings. Owing to the ketone binding to magnesium ions, the crystal structure exhibits a staggered ladder motif whereby a screw axis relates the chains; the staggered chains run along the *b*-axis. The 2D sheets are perpendicular to the *c*-axis.

**CaL**. Single-crystal X-ray analysis reveals that **CaL** crystallizes in the triclinic space group 1 (electronic supplementary material, table S3). The asymmetric unit contains one Ca^2+^ ion, one **L** molecule and two coordinated DMF molecules (figure 1*b*). There is only one type of calcium ion present throughout the crystal structure, in which each metal centre is octahedrally coordinated. The one-dimensional chain of metal ions extends along the *a*-axis, while the linkers connecting the metal chains propagate along the *b*-axis. The 2D sheets formed are hence perpendicular to the *c*-axis. Six oxygen atoms surround each calcium ion, whereby O(1) and O(2) come from the coordinated DMF molecules that bind in the axial positions. O(3)–O(6) are in the equatorial positions and come from carboxylate groups of four different **L** molecules. The other oxygen of each carboxylate binds to the next calcium ion along in the chain. The linker exists throughout the structure with the carboxylate groups oriented in a *trans*-conformation with respect to the plane of the central rings, in which one carboxylate group binds to two Ca^2+^ ions in one chain, while the other carboxylate group only binds to one Ca^2+^ ion in an adjacent chain.

**SrL**. Single-crystal X-ray analysis reveals that **SrL** crystallizes in the monoclinic space group *I*2/*a* (electronic supplementary material, table S4). The asymmetric unit contains one Sr^2+^ ion, one **L** molecule and one bridging water molecule on a special position (figure 1*c*). The interaction between metal cations and **L** anions gives rise to a structure consisting of 2D sheets which are perpendicular to the *c*-axis. There is only one type of strontium ion in the structure; however, they are in a staggered conformation whereby four different orientations propagate along the *a*-axis. The symmetry operation relating the strontium ions involves a rotation of 180° followed by a reflection (⊥ to *a*-axis) to move between Sr(1) and Sr(2). This is then followed by an inversion (between two Sr atoms), followed by a 90° rotation to move from Sr(2) to Sr(3). Sr(3) to Sr(4) requires a 180° rotation followed by reflection and Sr(4) to another Sr(1) requires inversion and 90° rotation. These operations can then be repeated to move along the staggered formation of strontium ions extending in the plane of the *a*-axis, while identical chains of strontium ions extend along the *b*-axis. Each strontium ion is six-coordinate. O(1) is the ketone oxygen from **L**, while O(2) is a carboxylate oxygen binding only to Sr(1). O(3) is an oxygen atom from a bridging water molecule. O(4) is a different carboxylate group binding to Sr(1). O(5) and O(6) are again from different carboxylate oxygens from two more linkers, each binding to Sr(1) and the identical Sr(1) in an adjacent chain. The linker differs from the magnesium structure in that it crystallizes with the carboxylate groups in a *cis*-conformation with respect to the plane of the central rings. The ketone oxygen binds to Sr(1) in an adjacent chain giving rise to the 2D sheets, while one carboxylate group binds solely to Sr(1). The one oxygen atom of the other carboxylate group binds to Sr(1), while the remaining oxygen atom binds to Sr(2) and Sr(2) in an adjacent chain. The bridging oxygen atom links Sr(2) and Sr(3).

**BaL**. Single-crystal X-ray analysis reveals that **BaL** crystallizes in the monoclinic space group *I*2/*a* (electronic supplementary material, table S5). The asymmetric unit contains one Ba^2+^ ion, one **L** molecule and one bridging H_2_O molecule on a special position (figure 1*d*). The structure consists of 2D sheets that are perpendicular to the *b*-axis. Identical chains of barium ions extend along the *b*-axis, while staggered chains of barium ions run along the *c*-axis. The symmetry elements that relate the barium ions in four different orientations are the same as in **SrL**. The coordination of the linker in the *cis*-conformational binding to the metal cations is isostructural to **SrL**.

**Figure 2.**
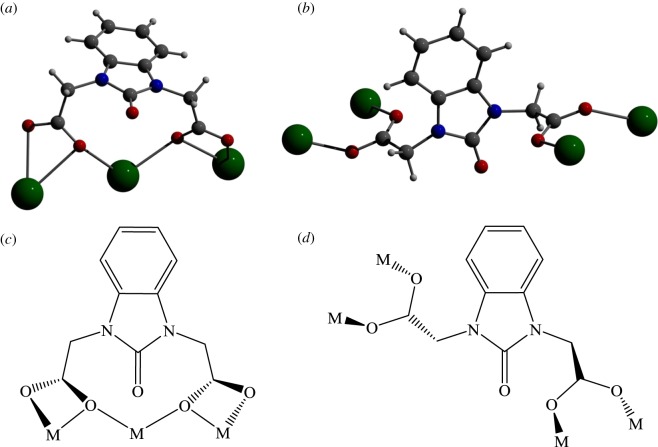
Flexibility of **L** allows for a *cis*- (*a,c*) or *trans*-conformation (*b*,*d*) to be adopted.

The four group 2 coordination polymers that have been synthesized all form 2D sheets and contain six-coordinate metal nodes. This was unexpected as we anticipated that the coordination number around the larger metal cations might be higher, particularly as there are several reported strontium and barium MOFs that display coordination numbers greater than seven [36,37]. The linker configuration of the carboxylate groups does differ between the smaller and larger group 2 metal structures, shown schematically in figure 2. In **MgL** and **CaL**, the linker carboxylate groups adopt a *trans*-conformation with respect to the plane of the central rings of the molecule and the carboxylate groups bind to different metal ions. Notably, in **SrL** and **BaL** structures the linker carboxylate groups adopt a *cis*-conformation and the two carboxylate groups both bind to the same metal node. This demonstrates the complex relationship between the linker flexibility and the metal ion used in the coordination polymer synthesis, and suggests that the larger cation size upon descending group 2 allows for chelate binding of the metal between two carboxylates and thus enables the linker to bind in the *cis*-conformation. Counterintuitively, the structures with larger cations thus result in a tighter mesh due to the capacity of both carboxylate groups in **L** to bind to the same metal. Magnesium and calcium cations are sufficiently smaller that the linker is incapable of binding in the *cis*-conformation, and therefore adopts a bridging *trans*-conformation.

### 3.3. Transition metal coordination polymers

**MnL**. Single-crystal X-ray analysis reveals that **MnL** crystallizes in the monoclinic space group *P*2*_1_*/*n* (electronic supplementary material, table S6). The asymmetric unit contains one Mn^2+^ ion, one **L** molecule, two water molecules and one coordinated DMF molecule (the methyl groups are disordered over two positions) (figure 1*e*). One of the water molecules hydrogen-bonds to the ketone oxygen of **L**. The crystal structure consists of 2D sheets that are perpendicular to the *b*-axis and contains identical manganese ions that have a slightly distorted octahedral geometry. In the equatorial plane, there are two water molecules in a *cis*-arrangement, a DMF molecule and an oxygen atom from the carboxylate group of **L**. Two linker molecules bind to the manganese ion in the axial positions through an oxygen of the carboxylate group. The other carboxylate groups bind to adjacent Mn^2+^ ions in the neighbouring chains, therefore linking the chains running along the *a*-axis.

**Cu_3_L_2_.** Single-crystal X-ray analysis reveals that **Cu_3_L_2_** crystallizes in the triclinic space group 1 (electronic supplementary material, table S7). The asymmetric unit contains three Cu^2+^ ions, two **L** molecules, two coordinated DMF molecules and two molecules of formate (figure 1*f*). There are three types of copper centres present in the coordination polymer. Cu(1) is square planar whereby two oxygen atoms come from the carboxylate groups of two different linker molecules and the other two are from DMF molecules coordinated *trans* to each other. Cu(2) and Cu(3) form a distorted paddle-wheel, whereby two oxygen atoms are from carboxylate groups of two different **L** molecules and the other two points of extension *trans* to each other are formate molecules. The axial substituent on the bottom of the paddle-wheel is a carboxylate from **L**, which binds through one oxygen atom to Cu(2), while the other binds to the square planar Cu(1) centre, thus bridging the two Cu environments and forming a staggered chain. Cu(3) forms the top half of the paddle-wheel in which **L** binds through the carboxylate group in the axial position. The alternating square planar Cu(1) and Cu(2)/Cu(3) paddle-wheel sequence extends along the *a*-axis, giving rise to 2D sheets that intersect the *b-* and *c*-axis. There are also two linker binding motifs throughout the crystal structure, both in the *trans*-conformation. In the first motif, the linker binds to Cu(2) and Cu(3) in a distorted paddle-wheel through both oxygen atoms of one carboxylate group, while the other carboxylate on the same linker binds to a square planar Cu(1) site via one oxygen atom, and the axial site of Cu(2) on a different paddle-wheel through the other. The second motif also involves one carboxylate binding to a distorted paddle-wheel, while the other carboxylate bridges the axial position of Cu(3) on the distorted paddle-wheel and the square planar Cu(1) site; however, the latter is achieved through only one oxygen atom on the carboxylate, leaving the other oxygen atom free.

**ZnL.** Single-crystal X-ray analysis reveals that **ZnL** crystallizes in the monoclinic space group *P*2*_1_* (electronic supplementary material, table S7). The asymmetric unit contains one Zn^2+^ ion, one **L** molecule and one coordinated DMF molecule (figure 1*g*). The structure exists as 2D sheets that are perpendicular to the *c*-axis. One-dimensional chains of zinc ions propagate along the *a*-axis, while the linker molecules extend along the *b*-axis. Only one type of Zn^2+^ centre is seen in the coordination polymer; two different orientations are present, related by a 180° rotation, thereby leading to formation of a zig-zag 2D sheet. Each metal centre is four-coordinate, in which four oxygen atoms are bound. O(1) is coordinated DMF, while O(2)–O(4) are oxygen atoms from carboxylate groups of three different linker molecules. As seen in **MnL**, the linker carboxylate groups adopt a *trans-*conformation throughout the structure. One carboxylate group of **L** bridges between two Zn^2+^ ions in a chain, while the other carboxylate group binds to a zinc ion in an adjacent chain.

All three of these coordination polymers form 2D sheets; however, there are notable differences in their structural geometries. **MnL** contains six-coordinate distorted octahedral metal centres, very commonly observed for Mn^2+^ complexes [38], and reminiscent of the group 2 metal coordination. **ZnL** was found to contain tetrahedrally coordinated metal centres, again commonly found in zinc(II) complexes and zinc metalloproteins [39,40]. **Cu_3_L_2_** is perhaps the most unusual of the synthesized coordination polymers due to the two types of copper present. Copper-based MOFs formed with carboxylates commonly contain a ‘paddle-wheel' unit formed during the self-assembly process, even in the presence of flexible carboxylate ligands [41]. However, in **Cu_3_L_2_** there are both distorted paddle-wheels and square planar coordination modes. Binuclear Cu(II) paddle-wheels are a common feature in some MOFs [42], but the preferred geometry for mononuclear Cu^2+^ transition metal complexes is square planar [43], making **Cu_3_L_2_** an interesting structure that contains both coordination geometries. In the three synthesized compounds discussed above, the linker adopts the *trans*-conformation, suggesting that the smaller size of the ions leads to this particular binding mode, or maybe even excludes the possibility of the *cis*-conformation.

The linker in the *cis*-conformation is only seen in **SrL** and **BaL**, whereas in all the transition metal structures and the smaller group 2 metal structures the *trans*-conformation is adopted. This suggests that the *cis* binding mode can only be achieved when metals with larger ionic radii are used in the synthesis. Bridging oxygen atoms from water molecules between metal centres are also only seen in the coordination polymers with larger metal cations (**SrL** and **BaL**) because the metal ions are a suitable distance apart to allow for such metal–oxygen–metal bonding. All of the coordination polymers exist in a 1 : 1 linker-to-metal ratio with only one type of metal centre observed, except for **Cu_3_L_2_** in which the ratio is 2 : 3 and two types of copper metal centres are present.

### 3.4. Bulk characterization

**Figure 3.**
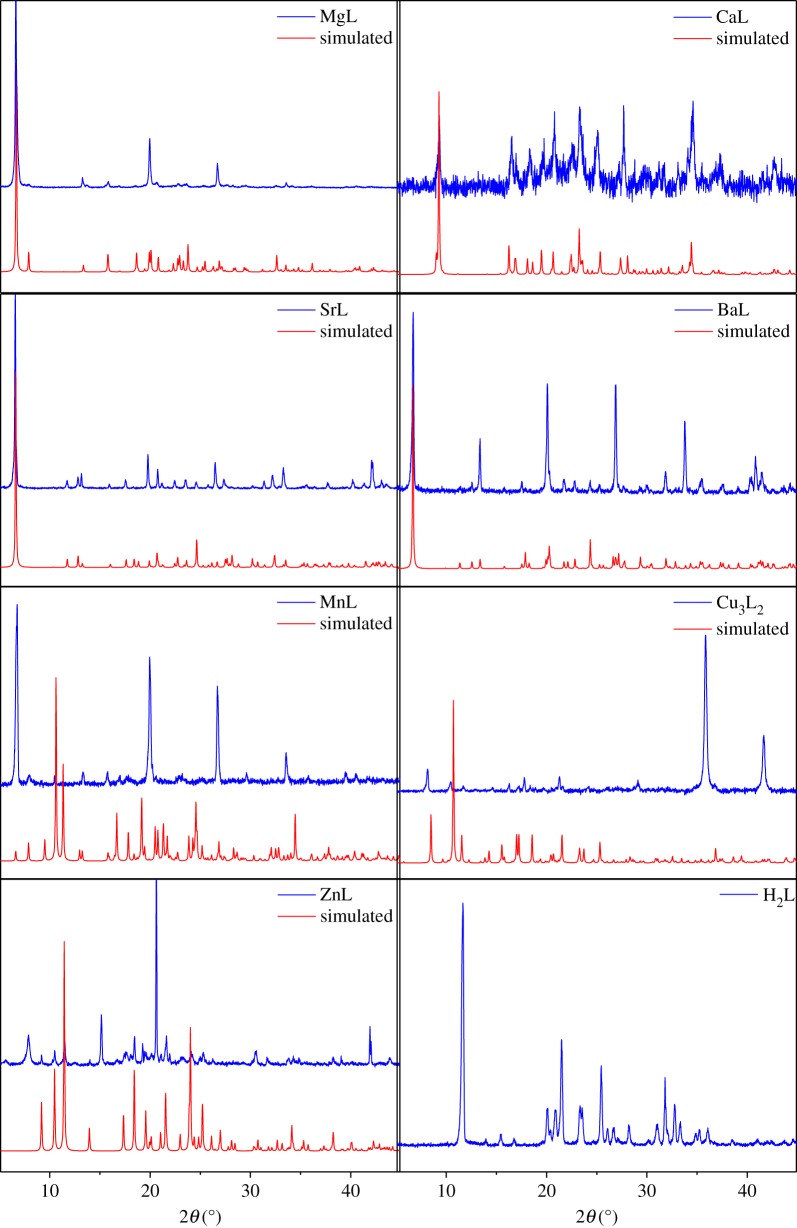
Simulated versus experimental PXRD patterns for the seven synthesized coordination polymers and **H_2_L** (the backgrounds of the **CaL** and **Cu_3_L_2_** experimental PXRD patterns have been corrected).

The above sections describe the single-crystal structures. We also studied the bulk crystalline powders of each sample by PXRD and TGA. Figure 3 compares the simulated PXRD pattern generated from the crystal structures with the experimental patterns for all seven products formed in the reactions that yielded X-ray-quality single crystals. All experimental PXRD patterns, except for **Cu_3_L_2_** and **ZnL**, were refined using a Pawley fit based on the simulated unit cell and space group [44]; they were refined to a reliable goodness of fit (*χ*^2^ is less than 4). Neither **Cu_3_L_2_** and **ZnL** are phase pure; copper oxide peaks are clearly seen at 36.45° and 42.38° 2*θ* in the relevant PXRD pattern, and spurious peaks (15.17°, 20.63° and 21.63°) in the zinc pattern can be attributed to zinc formate.

Electronic supplementary material, figure S1 shows the TGA plots for thermal decomposition of the coordination polymers and **H_2_L** in air. For **H_2_L**, three steps in the mass loss are seen; the first corresponds to the loss of coordinated water and the second step at 300°C can be attributed to the loss of both –CH_2_CO_2_H arms from the molecule. All the materials show a mass loss approximately 150**–**200°C. This is more than 100°C lower than the temperature at which the ligand alone loses the –CH_2_CO_2_H arms and, based also on the subsequent mass losses at higher temperature, is consistent with decarboxylation, i.e. loss of one or two CO_2_ molecules from each linker. The exact mechanism of thermal decarboxylation is not known in this instance, but is not uncommon for metal-coordinated carboxylates [45]. At higher temperatures, the group 2 metal coordination polymers decompose to a mixture of metal oxides and metal nitrides [46], making further quantitative analysis difficult.

## 4. Conclusion

In summary, we have synthesized seven coordination polymers using a flexible benzimidazolone diacetic acid linker with group 2 metals and first-row transition metals. All compounds were found to exist as 2D sheets with six-coordinate metal centres, except for **ZnL,** which showed tetrahedral coordination around the metal centre, and **Cu_3_L_2,_** which exhibited both distorted paddle-wheels and square planar coordinated Cu^2+^. The linker demonstrated its flexibility by coordinating with the –CH_2_CO_2_– arms in either a *cis-* or *trans-*conformation relative to the planar central portion of the molecule. The *cis*-conformation was only observed in **SrL** and **BaL**, and these were the only two structures that contained bridging water molecules between metal centres because the metal ions were sufficiently close together. The remaining coordination polymers, **MgL**, **CaL**, **MnL**, **Cu_3_L_2_** and **ZnL**, formed with the linker in the *trans*-conformation. **Cu_3_L_2_** was the only material to show a different linker-to-metal ratio, and also exhibited two different coordination modes of Cu^2+^. Finally, the bulk crystallinity was assessed by PXRD and TGA. The PXRD patterns of synthesized materials were in good agreement with the simulated data from the crystal structures; however, bulk **Cu_3_L_2_** and **ZnL** were found to be phase impure. TGA data showed that the coordination polymers were stable up to temperatures in the range of 150–200°C, after which decarboxylation occurs.

While the self-assembly of benzimidazolone diacetic acid in these coordination polymers demonstrates a range of carboxylate binding modes, two distinct structural motifs of the linker are observed. These *cis*/*trans* configurations are apparently dependent primarily on the size of the metal ion being coordinated. As a result of this study, we are now extending our limited investigation of transition metal complexes with **L** to include other biologically relevant metals such as iron and aluminium which, along with copper and zinc, are implicated in the formation of amyloid-β plaques in the brain [47].

## Supplementary Material

Supplementary Material

## Supplementary Material

BaL.cif

## Supplementary Material

CaL.cif

## Supplementary Material

Cu3L2.cif

## Supplementary Material

MgL.cif

## Supplementary Material

MnL.cif

## Supplementary Material

SrL.cif

## Supplementary Material

ZnL.cif

## Data accessibility

The datasets supporting this article have been uploaded as part of the electronic supplementary material. Our data are deposited at CONVERIS: http://doi.org/10.17035/d.2017.0040535794. CCDC 1566914-1566920 contains the supplementary crystallographic data for this paper. The data can be obtained free of charge from The Cambridge Crystallographic Data Centre via www.ccdc.cam.ac.uk/structures.
