# Supplementary material for: Investigating the geometrical preferences of a flexible benzimidazolone-based linker in the synthesis of coordination polymers
Source: R Soc Open Sci. 2017 Dec 6;4(12):171064. doi: 10.1098/rsos.171064 (PMC5750013; doi:10.1098/rsos.171064)
Supplement: Supplementary Material [file rsos171064supp1.docx]

**Investigating the geometrical preferences of a flexible benzimidazolone-based linker in the synthesis of** **coordination polymers.**

*Corey L. Jones^1^, Elizabeth A. Marsden^1^, Adam C. Nevin^1^, Benson Kariuki^1^, Mohan M. Bhadbhade^2^, Adam D. Martin*^3^ and Timothy L. Easun*^1^*

^1^School of Chemistry, Cardiff University, Main Building, Park Place, Cardiff, CF10 3AT

^2^School of Chemistry and Mark Wainwright Analytical Centre, The University of New South Wales, Sydney, NSW, 2052

^3^School of Chemistry, The Australian Centre for Nanomedicine and the ARC Centre of Excellence in Convergent Bio-Nano Science, The University of New South Wales, Sydney, NSW, 2052

**Supporting Information**

**Supporting Information**

**Part 1: Experimental procedures**

**Table of contents**

Synthesis of **H_2_L**…………………………………………………….........................page SI - 3

Synthesis of single crystals of coordination polymers……………..……...………...page SI - 4

Table S1………………………………………………………………………..……page SI - 5

Figure S1………………………………………………………………………….…page SI - 9

**Part 2: Crystallographic Information**

**Table of contents**

Table S2…….………………………………………………………...……............page SI - 10

Table S3..….………………………………………………………...……..............page SI - 11

Table S4…….………………………………………………………...……............page SI - 12 Table S5…….………………………………………………………...……............page SI - 13

Table S6…….………………………………………………………...……............page SI - 14

Table S7…….………………………………………………………...……............page SI - 15

Table S8…….………………………………………………………...……............page SI - 16

**Synthesis of H_2_L**

**Synthesis of** **benzimidazolone.** Phenylenediamine (5.4 g, 0.05 mol), urea (3.4 g, 0.057 mol) and ethylene glycol (25 mL) were stirred at 135 °C for 1 hour; a colour change from orange to red-orange was seen. The solution was then stirred overnight (18 hours) at 170 °C. The dark red solution was cooled down to 40-50 °C before the addition of 95% ethanol (5 mL, 0.08 mol) which was stirred for 10 minutes and then distilled water (20 mL) was added. The brown precipitate was washed with successive batches of water and 95% ethanol (~ 1 L), before drying under vacuum to give benzimidazolone as a pale brown solid (3.54 g, 60%). **^1^H NMR (400 MHz, DMSO):** δ_H_ = 10.56 (s, 2H), 6.91 (m, 4H). **^13^C NMR (101 MHz, DMSO):** δ_C_ = 155.2, 129.6, 120.37, 108.4. **IR:** ν_max_/ cm^-1^ **=** 2895, 1630, 1360, 1196, 725, 714, 590. **HRMS (ES^+^):** *m/z* **=** 134.0479; calculated 134.0480 for [C_7_H_6_N_2_O]^+^.

**Synthesis of** **benzimidazolone diethyl acetate ester.** NaH (60% in mineral oil) (746 mg, 12.43 mmol) was added to hexane (20 mL) under N_2_. After stirring for one minute, excess hexane was removed, before repeating once more. Anhydrous DMF (50 mL) was added, and the brown solution was stirred for 2 minutes. Benzimidazolone (1 g, 7.46 mmol) was added slowly and stirred for 30 minutes to form a grey cloudy solution. After cooling to 0 °C, ethyl bromoacetate (1.8 mL, 16.41 mmol) was added dropwise, resulting in a brown solution, which was stirred for a further 90 minutes. Keeping the reaction mixture at 0 °C, methanol was added to quench the reaction giving a yellow solution. The addition of water (150 mL) formed a white precipitate. The white solid was extracted into ethyl acetate (150 mL), washed with water (3 x 100 mL) and subsequently dried under reduced pressure to give benzimidazolone diethyl acetate ester as an off-white solid (1.51 g, 66%). The solid was purified by column chromatography (3:2 hexane:ethyl acetate). **^1^H NMR (400 MHz, CDCl_3_):** δ_H_ = 7.11 (dd, *J* = 5.80, 3.20 Hz, 2H), 6.91 (dd, *J* = 5.70, 3.20 Hz, 2H), 4.64 (s, 4H), 4.23 (q, *J* = 7.1 Hz, 4H), 1.27 (t, *J* = 7.1 Hz, 6H). **^13^C NMR (101 MHz, CDCl_3_):** δ_C_ = 167.8, 154.1, 129.3, 122.1, 108.0, 62.0, 42.6, 14.3. **IR:** ν_max_/ cm^-1^ **=** 1724, 1429, 1372, 1210, 750. **HRMS (ES^+^):** *m/z* **=** 307.1285; calculated 307.1294 for [C_15_H_18_N_2_O_5_]^+^.

**Synthesis of** **benzimidazolone diacetic acid (H_2_L).** Benzimidazolone diethyl acetate ester (1.25 g, 4.09 mmol) was dissolved in methanol (50 mL) and THF (50 mL). 2 M NaOH (50 mL, 100 mmol) was added and the solution was stirred at 30 °C for 24 hours. The solvents were removed under reduced pressure, before water was added (20 mL) and the yellow solution was acidified with 6 M HCl (aq). The solution turned colourless at approximately pH 6, and a white precipitate formed at pH 2. The precipitate was filtered to give benzimidazolone diacetic acid as a white solid (0.574 g, 56%). **^1^H NMR (400 MHz, DMSO):** δ_H_ = 7.16 (dd, *J* = 5.80, 3.20 Hz, 2H), 7.06 (dd, *J* = 5.80, 3.20 Hz, 2H), 4.62 (s, 4H). **^13^C NMR (101 MHz, DMSO):** δ_C_ = 169.5, 153.5, 129.1, 121.2, 108.3, 42.0. **IR:** ν_max_/ cm^-1^ **=** 3343, 1676, 1437, 1265, 750, 598. **HRMS (ES^+^):** *m/z* = 251.0677; calculated 251.0668 for [C_11_H_10_N_2_O_5_]^+^.

**Synthesis of single crystal of coordination polymers**

**Synthesis of MgL.** Mg(NO_3_)_2_·6H_2_O (31 mg, 0.12 mmol) and **H_2_L** (10 mg, 0.04 mmol) were dissolved in DMF (2 mL) in an 8 mL Wheaton vial. Ethanol (0.5 mL) and water (0.2 mL) were added to the solution which was then sealed and heated at 80 °C for 2 days to give colourless crystals. The crystals were used to seed a second reaction, under the same conditions, yielding larger colourless crystals. **IR:** ν_max_/ cm^-1^ **=** 3211, 2160, 1709, 1651, 1585, 1495, 1447, 1387, 1342, 1312, 1207, 1184, 1057, 1011, 928, 854, 754, 704, 655.

**Synthesis of CaL.** Ca(NO_3_)_2_·4H_2_O (71 mg, 0.30 mmol) and **H_2_L** (25 mg, 0.10 mmol) were dissolved in DMF (2.5 mL) in an 8 mL Wheaton vial. Formic acid (11 µL) was added to the solution which was then sealed and heated at 100 °C for 24 h yielding colourless crystals. **IR:** ν_max_/ cm^-1^ = 3450, 2300, 1757, 1689, 1667, 1593, 1495, 1439, 1387, 1315, 1300, 1207, 1182, 1045, 1012, 935, 914, 818, 739, 704, 662.

**Synthesis of SrL.** Sr(NO_3_)_2_ (25 mg, 0.12 mmol) and **H_2_L** (10 mg, 0.04 mmol) were dissolved in DMF (2.5 mL) in an 8 mL Wheaton vial. 0.4 M HCl (1 mL) was added to the solution which was then sealed and heated at 80 °C for 24 h yielding colourless crystals. **IR:** ν_max_/ cm^-1^ **=** 3356, 3000, 2910, 2790, 1694, 1585, 1497, 1437, 1404, 1379, 1312, 1202, 1171, 1063, 1011, 912, 833, 791, 706, 652.

**Synthesis of BaL.** Ba(NO_3_)_2_ (31 mg, 0.12 mmol) and **H_2_L** (10 mg, 0.04 mmol) were dissolved in DMF (2.5 mL) in an 8 mL Wheaton vial. 0.4 M HCl (1 mL) was added to the solution which was then sealed and heated at 80 °C for 24 h yielding colourless crystals. **IR:** ν_max_/ cm^-1^ **=** 3356, 2990, 2779, 1694, 1595, 1495, 1402, 1383, 1312, 1171, 1011,787, 739, 706, 610.

**Synthesis of MnL** Mn(NO_3_)_2_·4H_2_O (30 mg, 0.12 mmol) and **H_2_L** (10 mg, 0.04 mmol) were dissolved in DMF (2.5 mL) in an 8 mL Wheaton vial. 0.2 M HCl (1 mL) was added to the solution which was then sealed and heated at 80 °C for 2 days yielding colourless crystals. **IR:** ν_max_/ cm^-1^ **=** 3410, 3085, 2990, 1667, 1574, 1495, 1445, 1383, 1304, 1206, 1188, 1011, 850, 748, 723, 658.

**Synthesis of Cu_3_L_2_.** Cu(NO_3_)_2_·3H_2_O (73 mg, 0.30 mmol) and **H_2_L** (25 mg, 0.10 mmol) were dissolved in DMF (2.5 mL) in an 8 mL Wheaton vial. Formic acid (11 µL) was added to the solution which was then sealed and heated at 90 °C for 24 h yielding green crystals. **IR:** ν_max_/ cm^-1^ = 3211, 2355, 2183, 2018, 1676, 1570, 1495, 1435, 1385, 1315, 1269, 1204, 1177, 1057, 1013, 918, 756, 710, 619.

**Synthesis of ZnL.** Zn(NO_3_)_2_·6H_2_O (90 mg, 0.30 mmol) and **H_2_L** (25 mg, 0.10 mmol) were dissolved in DMF (2.5 mL) in an 8 mL Wheaton vial. Formic acid (11 µL) was added to the solution which was then sealed and heated at 80 °C for 24 h yielding colourless product. Single crystals were obtained by using the same conditions and heating the reaction to 100 °C for 24 h. I**R:** ν_max_/ cm^-1^ = 3350, 2191, 2004, 1667, 1632, 1497, 1580, 1497, 1456, 1385, 1310, 1296, 1275, 1206, 1188, 1053, 1015, 924, 847, 773, 727, 712, 685, 664.

**Table S1.** Attempted reaction conditions for coordination polymer synthesis.

| **Metal salt** | **Mass of L / mg** | **Mass of M / mg** | **DMF / mL** | **Modulator / co-solvent** | **Temp. / °C** |
| --- | --- | --- | --- | --- | --- |
| Mg(NO_3_)_2_.6H_2_O | 10 | 29 | 2.5 | 0.1 M HCl, 0.1 mL | 90 |
|  |  |  |  | 0.1 M HCl, 0.5 mL |  |
|  |  |  |  | 0.1 M HCl, 1 mL |  |
|  |  |  |  | 0.5 M HCl, 0.1 mL |  |
|  |  |  |  | 0.5 M HCl, 0.5 mL |  |
|  |  |  |  | 0.5 M HCl, 1 mL |  |
|  |  |  |  | 1 M HCl, 0.1 mL |  |
|  |  |  |  | 1 M HCl, 0.5 mL |  |
|  |  |  |  | 1 M HCl, 1 mL |  |
|  |  |  |  | 2 M HCl, 0.1 mL |  |
|  |  |  |  | 2 M HCl, 0.5 mL |  |
|  |  |  |  | 2 M HCl, 1 mL |  |
|  |  |  |  | 6 M HCl, 1 drop |  |
|  |  |  |  | 6 M HCl, 3 drops |  |
|  |  |  |  | 6 M HCl, 5 drops |  |
|  |  |  |  | Formic acid, 5 μL |  |
|  |  |  |  | Formic acid, 10 μL |  |
|  |  |  |  | Formic acid, 15 μL |  |
|  |  |  |  | Formic acid, 20 μL |  |
|  |  |  |  | Formic acid, 25 μL |  |
|  |  |  |  | Formic acid, 30 μL |  |
|  |  |  |  | Formic acid, 35 μL |  |
|  |  |  |  | Formic acid, 40 μL |  |
|  |  |  |  | 0.1 M HCl, 0.5 mL | 80 |
|  |  |  |  | 0.5 M HCl, 0.5 mL |  |
|  |  |  |  | 2 M HCl, 1 mL |  |
|  |  |  |  | 0.1 M HCl, 0.1 mL |  |
|  |  |  |  | 0.1 M HCl, 0.25 mL |  |
|  |  |  |  | 0.5 M HCl, 0.1 mL |  |
|  |  |  |  | 0.5 M HCl, 0.25 mL |  |
|  |  |  |  | 1 M HCl, 0.1 mL |  |
|  |  |  |  | 1 M HCl, 0.25 mL |  |
|  |  |  |  | Formic acid, 5 μL |  |
|  |  |  |  | Formic acid, 10 μL |  |
|  |  |  |  | Formic acid, 15 μL |  |
|  |  |  |  | Formic acid, 20 μL |  |
|  |  | 31 | 1 | 0.1 M HCl, 0.5 mL |  |
|  |  |  |  | Formic acid, 4.5 μL |  |
|  |  | 31 | 2.5 | - |  |
|  |  |  | 2 | 0.5 mL EtOH, 3 drops H_2_O |  |
|  |  |  |  | 0.6 mL EtOH, 0.1 mL H_2_O |  |
|  |  |  |  | 0.6 mL EtOH, 0.1 mL H_2_O |  |
|  |  |  |  | 0.5 mL EtOH, 0.1 mL H_2_O |  |
|  |  |  |  | 0.4 mL EtOH, 0.1 mL H_2_O |  |
|  |  |  |  | 0.3 mL EtOH, 0.1 mL H_2_O |  |
|  |  |  |  | 0.5 mL EtOH |  |
|  |  |  |  | 0.5 mL EtOH, 0.05 mL H_2_O |  |
|  |  |  |  | 0.5 mL EtOH, 0.15 mL H_2_O |  |
|  |  |  |  | 0.2 mL EtOH, 0.15 mL H_2_O |  |

| **Metal salt** | **Mass of L / mg** | **Mass of M / mg** | **DMF / mL** | **Modulator / co-solvent** | **Temp. / °C** |
| --- | --- | --- | --- | --- | --- |
| Mg(NO_3_)_2_.6H_2_O | 25 | 78 | 2.5 | Formic acid, 11 µL | 80 |
|  |  |  |  | Formic acid, 11 µL | 90 |
|  |  |  |  | Formic acid, 11 µL | 100 |
| Ca(NO_3_)_2_.4H_2_O | 25 | 72 | 2.5 | Formic acid, 11 μL | 80 |
|  |  |  |  | Formic acid, 11 μL | 90 |
|  |  |  |  | Formic acid, 11 μL | 100 |
| Sr(NO_3_)_2_ | 10 | 25 | 2.5 | 0.1 M HCl, 0.1 mL | 90 |
|  |  |  |  | 0.1 M HCl, 0.5 mL |  |
|  |  |  |  | 0.1 M HCl, 1 mL |  |
|  |  |  |  | 0.5 M HCl, 0.1 mL |  |
|  |  |  |  | 0.5 M HCl, 0.5 mL |  |
|  |  |  |  | 0.5 M HCl, 1 mL |  |
|  |  |  |  | 1 M HCl, 0.1 mL |  |
|  |  |  |  | 1 M HCl, 0.5 mL |  |
|  |  |  |  | 1 M HCl, 1 mL |  |
|  |  |  |  | 2 M HCl, 0.1 mL |  |
|  |  |  |  | 2 M HCl, 0.5 mL |  |
|  |  |  |  | 2 M HCl, 1 mL |  |
|  |  |  |  | 6 M HCl, 1 drop |  |
|  |  |  |  | 6 M HCl, 3 drops |  |
|  |  |  |  | 6 M HCl, 5 drops |  |
|  |  |  |  | Formic acid, 5 μL |  |
|  |  |  |  | Formic acid, 10 μL |  |
|  |  |  |  | Formic acid, 15 μL |  |
|  |  |  |  | Formic acid, 20 μL |  |
|  |  |  |  | Formic acid, 25 μL |  |
|  |  |  |  | Formic acid, 30 μL |  |
|  |  |  |  | Formic acid, 35 μL |  |
|  |  |  |  | Formic acid, 40 μL |  |
|  |  |  |  | 0.1 M HCl, 0.1 mL | 80 |
|  |  |  |  | 0.1 M HCl, 0.5 mL |  |
|  |  |  |  | 0.1 M HCl, 1 mL |  |
|  |  |  |  | 0.5 M HCl, 0.1 mL |  |
|  |  |  |  | 0.5 M HCl, 0.5 mL |  |
|  |  |  |  | 0.5 M HCl, 1 mL |  |
|  |  |  |  | 1 M HCl, 0.1 mL |  |
|  |  |  |  | 1 M HCl, 0.5 mL |  |
|  |  |  |  | 1 M HCl, 1 mL |  |
|  |  |  |  | 2 M HCl, 0.1 mL |  |
|  |  |  |  | 2 M HCl, 0.5 mL |  |
|  |  |  |  | 2 M HCl, 1 mL |  |
|  |  |  |  | Formic acid, 5 μL |  |
|  |  |  |  | Formic acid, 10 μL |  |
|  |  |  |  | Formic acid, 15 μL |  |
|  |  |  |  | Formic acid, 20 μL |  |
|  |  |  |  | 0.4 M HCl, 0.5 mL |  |
|  |  |  |  | 0.4 M HCl, 0.75 mL |  |
|  |  |  |  | 0.4 M HCl, 1 mL |  |
|  |  |  |  | 0.4 M HCl, 1.25 mL |  |
|  |  |  |  | 0.5 M HCl, 0.5 mL |  |
|  | 10 | 25 | 2.5 | 0.5 M HCl, 0.75 mL |  |
|  |  |  |  | 0.5 M HCl, 1 mL |  |

| **Metal salt** | **Mass of L / mg** | **Mass of M / mg** | **DMF / mL** | **Modulator / co-solvent** | **Temp. / °C** |
| --- | --- | --- | --- | --- | --- |
| Sr(NO_3_)_2_ | 10 | 25 | 2.5 | 0.5 M HCl, 1.25 mL | 80 |
|  |  |  |  | 0.6 M HCl, 0.5 mL |  |
|  |  |  |  | 0.6 M HCl, 0.75 mL |  |
|  |  |  |  | 0.6 M HCl, 1 mL |  |
|  |  |  |  | 0.6 M HCl, 1.25 mL |  |
|  | 25 | 64 | 2.5 | Formic acid, 11 μL | 80 |
|  |  |  |  | Formic acid, 11 μL | 90 |
|  |  |  |  | Formic acid, 11 μL | 100 |
| Ba(NO_3_)_2_ | 10 | 31 | 2.5 | 0.4 M, 1 mL | 80 |
|  |  |  |  | 0.5 M, 1.25 mL |  |
|  | 25 | 79 | 2.5 | Formic acid, 11 μL | 80 |
|  |  |  |  | Formic acid, 11 μL | 90 |
|  |  |  |  | Formic acid, 11 μL | 100 |
| Cr(NO_3_)_2_.9H_2_O | 10 | 48 | 2.5 | 0.1 M HCl, 0.5 mL | 80 |
|  |  |  |  | 0.1 M HCl, 0.75 mL |  |
|  |  |  |  | 0.1 M HCl, 1 mL |  |
|  |  |  | 4 | 1 M HCl, 0.05 mL | 120 |
|  |  |  |  | 1 M HCl, 0.1 mL |  |
|  |  |  | 2.5 | Formic acid, 5 μL | 80 |
|  |  |  |  | Formic acid, 10 μL |  |
|  |  |  |  | Formic acid, 15 μL |  |
|  |  |  |  | Formic acid, 20 μL |  |
| Mn(NO_3_)_2_.4H_2_O | 10 | 30 | 2.5 | 0.1 M HCl, 0.5 mL |  |
|  |  |  |  | 0.1 M HCl, 0.75 mL |  |
|  |  |  |  | 0.1 M HCl, 1 mL |  |
|  |  |  |  | 0.1 M HCl, 1.25 mL |  |
|  |  |  |  | Formic acid, 5 μL |  |
|  |  |  |  | Formic acid, 10 μL |  |
|  |  |  |  | Formic acid, 15 μL |  |
|  |  |  |  | Formic acid, 20 μL |  |
|  |  |  |  | 0.5 M HCl, 1 mL |  |
|  |  |  | 4 | 1 M HCl, 0.05 mL | 120 |
|  |  |  |  | 1 M HCl, 0.1 mL |  |
|  |  |  | 2 | 0.5 mL EtOH, 0.2 mL H_2_O | 80 |
| Fe(NO_3_)_2_.9H_2_O | 10 | 49 | 2.5 | 0.1 M HCl, 0.5 mL |  |
|  |  |  |  | 0.1 M HCl, 0.75 mL |  |
|  |  |  |  | 0.1 M HCl, 1 mL |  |
|  |  |  | 4 | 1 M HCl, 0.05 mL | 120 |
|  |  |  |  | 1 M HCl, 0.1 mL |  |
| Co(NO_3_)_2_.6H_2_O | 10 | 35 | 2.5 | 0.1 M HCl, 0.5 mL | 80 |
|  |  |  |  | 0.1 M HCl, 0.75 mL |  |
|  |  |  |  | 0.1 M HCl, 1 mL |  |
|  |  |  | 4 | 1 M HCl, 0.05 mL | 120 |
|  |  |  |  | 1 M HCl, 0.1 mL |  |
| Ni(NO_3_)_2_.6H_2_O | 10 | 35 | 2.5 | 0.1 M HCl, 0.1 mL | 90 |
|  |  |  |  | 0.1 M HCl, 0.5 mL |  |
|  |  |  |  | 0.1 M HCl, 1 mL |  |
|  |  |  |  | 0.5 M HCl, 0.1 mL |  |
|  |  |  |  | 0.5 M HCl, 0.5 mL |  |
|  |  |  |  | 0.5 M HCl, 1 mL |  |
|  |  |  |  | 1 M HCl, 0.1 mL |  |
|  |  |  |  | 1 M HCl, 0.5 mL |  |

| **Metal salt** | **Mass of L / mg** | **Mass of M / mg** | **DMF / mL** | **Modulator / co-solvent** | **Temp. / °C** |
| --- | --- | --- | --- | --- | --- |
| Ni(NO_3_)_2_.6H_2_O | 10 | 35 | 2.5 | 1 M HCl, 1 mL | 90 |
|  |  |  |  | 2 M HCl, 0.1 mL |  |
|  |  |  |  | 2 M HCl, 0.5 mL |  |
|  |  |  |  | 2 M HCl, 1 mL |  |
|  |  |  |  | 6 M HCl, 1 drop |  |
|  |  |  |  | 6 M HCl, 3 drops |  |
|  |  |  |  | 6 M HCl, 5 drops |  |
|  |  |  | 4 | 1 M HCl, 0.05 mL | 120 |
|  |  |  |  | 1 M HCl, 0.1 mL |  |
| Cu(NO_3_)_2_.3H_2_O | 10 | 29 | 2.5 | 0.1 M HCl, 0.1 mL | 90 |
|  |  |  |  | 0.1 M HCl, 0.5 mL |  |
|  |  |  |  | 0.1 M HCl, 1 mL |  |
|  |  |  |  | 0.5 M HCl, 0.1 mL |  |
|  |  |  |  | 0.5 M HCl, 0.5 mL |  |
|  |  |  |  | 0.5 M HCl, 1 mL |  |
|  |  |  |  | 1 M HCl, 0.1 mL |  |
|  |  |  |  | 1 M HCl, 0.5 mL |  |
|  |  |  |  | 1 M HCl, 1 mL |  |
|  |  |  |  | 2 M HCl, 0.1 mL |  |
|  |  |  |  | 2 M HCl, 0.5 mL |  |
|  |  |  |  | 2 M HCl, 1 mL |  |
|  |  |  |  | 6 M HCl, 1 drop |  |
|  |  |  |  | 6 M HCl, 3 drops |  |
|  |  |  |  | 6 M HCl, 5 drops |  |
|  | 25 | 73 | 2.5 | Formic acid, 11 μL | 80 |
|  |  |  |  | Formic acid, 11 μL | 90 |
|  |  |  |  | Formic acid, 11 μL | 100 |
| Zn(NO_3_)_2_.6H_2_O | 25 | 90 | 2.5 | Formic acid, 11 μL | 80 |
|  |  |  |  | Formic acid, 11 μL | 90 |
|  |  |  |  | Formic acid, 11 μL | 100 |
| Al(NO_3_)_2_.9H_2_O | 25 | 114 | 2.5 | Formic acid, 11 μL | 80 |
|  |  |  |  | Formic acid, 11 μL | 90 |
|  |  |  |  | Formic acid, 11 μL | 100 |
| In(NO_3_)_2_.H_2_O | 25 | 97 | 2.5 | Formic acid, 11 μL | 80 |
|  |  |  |  | Formic acid, 11 μL | 90 |
|  |  |  |  | Formic acid, 11 μL | 100 |

**Figure S1:** TGA plots for **L** and the seven synthesised coordination polymers.


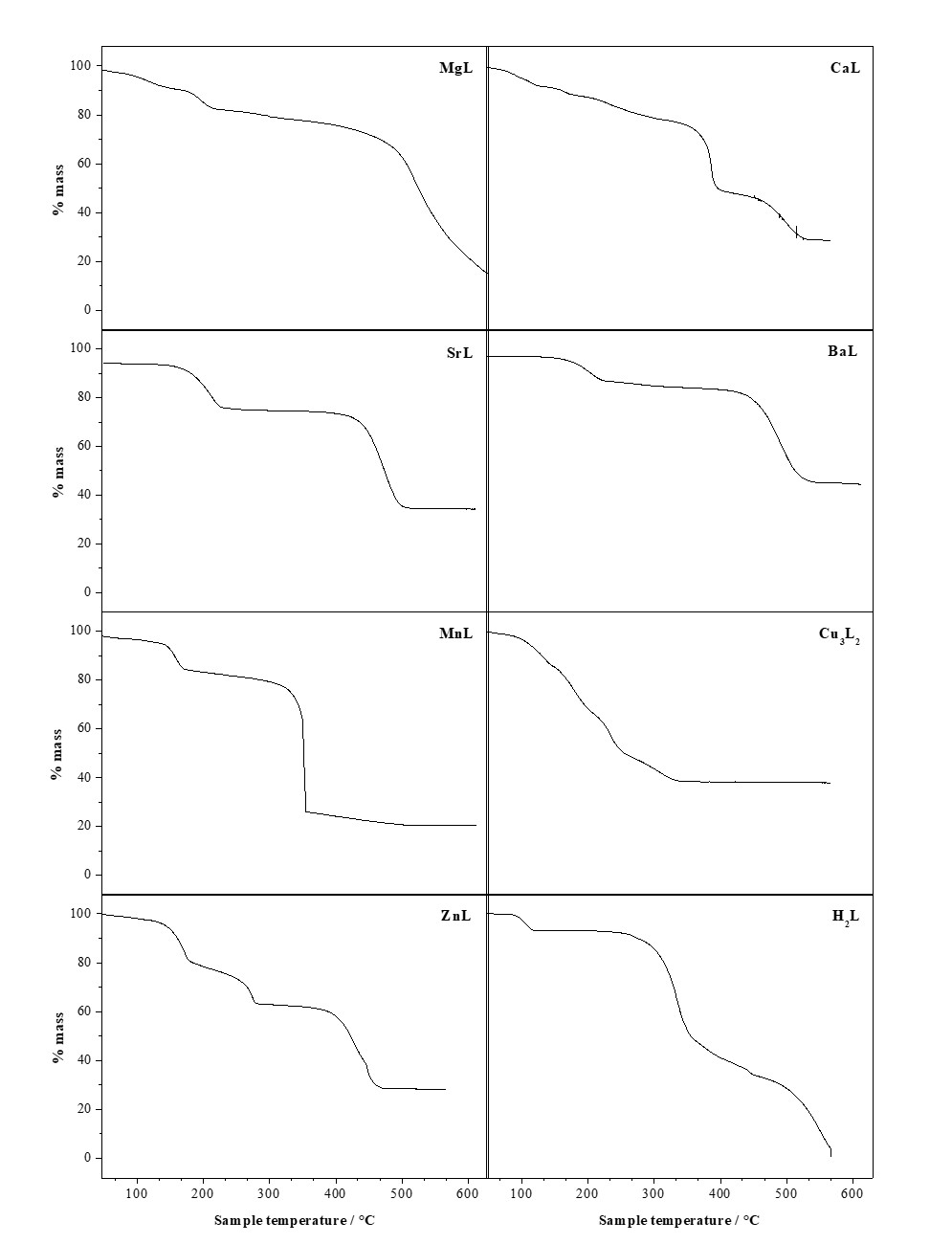


The lack of phase purity, seen in the PXRD patterns, for **Cu_3_L_2_** and **ZnL** means that quantitative conclusions on mass loss cannot be made.

**Table S2.** Single crystal data and structure refinement details for **MgL**.

| Chemical formula | (C_53_H_61_N_11_O_27_Mg_4_) |
| --- | --- |
| *M*_r_ (g mol^-1^) | 1381.36 |
| Crystal system, space group | [Triclinic](file:///C:\Users\Corey\Dropbox%20(Personal)\RS%20Open%20Science%20Paper%20June%202017\te1709c%20_space_group_crystal_system), [*P*](file:///C:\Users\Corey\Dropbox%20(Personal)\RS%20Open%20Science%20Paper%20June%202017\te1709c%20_space_group_name_H-M_alt)-1 |
| Temperature (K) | 150 |
| *a, b, c* (Å) | 4.8920(5), 11.6429(10), 13.6762(14) |
| *α, β, γ (°)* | 101.100(8), 96.884(8), 100.184(8) |
| *V* (Å^3^) | 742.74(13) |
| Z | 2 |
| Radiation type | Mo *K*α |
| μ (mm^-1^) | 0.16 |
| Crystal size (mm) | 0.34 x 0.10 x 0.06 |
| Absorption correction | Multi-scan |
| T_min_, T_max_ | 0.482, 1.000 |
| No. of observed measured, independent and observed [*I* > 2σ(*I*)] reflections | 6310, 3459, 1975 |
| *R*_int_ | 0.050 |
| (sinθ/λ)max (Å^-1^) | 0.694 |
| *R*[F2 > 2σ(F2)], ωR(F^2^), S | 0.110, 0.342, 1.03 |
| No. of reflections | 3459 |
| No. of parameters | 218 |
| H-atom treatment | H-atom parameters constrained |
| Δρ_max_, Δρ_min_ (e Å^-3^) | 1.72, -0.75 |

**Table S3.** Single crystal data and structure refinement details for **CaL**.

| Chemical formula | ([C_17_H_22_N_4_O_7_](file:///C:\Users\Corey\Dropbox%20(Personal)\RS%20Open%20Science%20Paper%20June%202017\tle1604%20_chemical_formula_moiety)Ca) |
| --- | --- |
| *M*_r_ (g mol^-1^) | 434.46 |
| Crystal system, space group | [Triclinic](file:///C:\Users\Corey\Dropbox%20(Personal)\RS%20Open%20Science%20Paper%20June%202017\te1709c%20_space_group_crystal_system), [*P*](file:///C:\Users\Corey\Dropbox%20(Personal)\RS%20Open%20Science%20Paper%20June%202017\te1709c%20_space_group_name_H-M_alt)-1 |
| Temperature (K) | 150 |
| *a, b, c* (Å) | 9.3304(10), 11.2571(12), 11.4390(12) |
| *α, β, γ (°)* | 108.146(9), 111.750(10), 103.744(9) |
| *V* (Å^3^) | 971.9(2) |
| Z | 2 |
| Radiation type | Mo *K*α |
| μ (mm^-1^) | 0.37 |
| Crystal size (mm) | 0.41 x 0.08 x 0.05 |
| Absorption correction | Gaussian |
| T_min_, T_max_ | 0.985, 0.997 |
| No. of observed measured, independent and observed [*I* > 2σ(*I*)] reflections | 7579, 4611, 2824 |
| *R*_int_ | 0.041 |
| (sinθ/λ)max (Å^-1^) | 0.698 |
| *R*[F2 > 2σ(F2)], ωR(F^2^), S | 0.064, 0.145, 1.06 |
| No. of reflections | 4611 |
| No. of parameters | 266 |
| H-atom treatment | H-atom parameters constrained |
| Δρ_max_, Δρ_min_ (e Å^-3^) | 0.37, -0.42 |

**Table S4.** Single crystal data and structure refinement details for **SrL**.

| Chemical formula | ([C](file:///C:\Users\Corey\Dropbox%20(Personal)\RS%20Open%20Science%20Paper%20June%202017\tle1708c%20_chemical_formula_moiety)_22_[H_16_N_4_O](file:///C:\Users\Corey\Dropbox%20(Personal)\RS%20Open%20Science%20Paper%20June%202017\tle1708c%20_chemical_formula_moiety)_11_[Sr](file:///C:\Users\Corey\Dropbox%20(Personal)\RS%20Open%20Science%20Paper%20June%202017\tle1708c%20_chemical_formula_moiety)_2_) |
| --- | --- |
| *M*_r_ (g mol^-1^) | 687.62 |
| Crystal system, space group | [Monoclinic](file:///C:\Users\Corey\Dropbox%20(Personal)\RS%20Open%20Science%20Paper%20June%202017\te1709c%20_space_group_crystal_system), *I*2/*a* |
| Temperature (K) | 150 |
| *a, b, c* (Å) | 17.1449(6), 4.8951(2), 26.8363(14) |
| *β (°)* | 95.495(4) |
| *V* (Å^3^) | 2241.93(18) |
| Z | 8 |
| Radiation type | Cu *K*α |
| μ (mm^-1^) | 6.96 |
| Crystal size (mm) | 0.14 x 0.05 x 0.02 |
| Absorption correction | Gaussian |
| T_min_, T_max_ | 0.838, 1.000 |
| No. of observed measured, independent and observed [*I* > 2σ(*I*)] reflections | 4021, 2192, 1849 |
| *R*_int_ | 0.049 |
| (sinθ/λ)max (Å^-1^) | 0.623 |
| *R*[F2 > 2σ(F2)], ωR(F^2^), S | 0.045, 0.114, 1.02 |
| No. of reflections | 2192 |
| No. of parameters | 169 |
| H-atom treatment | H-atom parameters constrained |
| Δρ_max_, Δρ_min_ (e Å^-3^) | 0.85, -1.30 |

**Table S5.** Single crystal data and structure refinement details for **BaL**.

| Chemical formula | ([C_22_H_16_N_4_O_11_Ba_2_)](file:///C:\Users\Corey\Dropbox%20(Personal)\RS%20Open%20Science%20Paper%20June%202017\tle1708c%20_chemical_formula_moiety) |
| --- | --- |
| *M*_r_ (g mol^-1^) | 787.07 |
| Crystal system, space group | [Monoclinic](file:///C:\Users\Corey\Dropbox%20(Personal)\RS%20Open%20Science%20Paper%20June%202017\te1709c%20_space_group_crystal_system), *I*2/*a* |
| Temperature (K) | 150 |
| *a, b, c* (Å) | 26.6103(14), 5.0441(2), 17.9012(9) |
| *β (°)* | 96.211(4) |
| *V* (Å^3^) | 2388.7(2) |
| Z | 8 |
| Radiation type | Cu *K*α |
| μ (mm^-1^) | 26.13 |
| Crystal size (mm) | 0.33 x 0.05 x 0.03 |
| Absorption correction | Multi-scan |
| T_min_, T_max_ | 0.485, 1.000 |
| No. of observed measured, independent and observed [*I* > 2σ(*I*)] reflections | 6650, 2392, 2054 |
| *R*_int_ | 0.071 |
| (sinθ/λ)max (Å^-1^) | 0.625 |
| *R*[F2 > 2σ(F2)], ωR(F^2^), S | 0.074, 0.217, 1.05 |
| No. of reflections | 2392 |
| No. of parameters | 130 |
| H-atom treatment | H-atom parameters constrained |
| Δρ_max_, Δρ_min_ (e Å^-3^) | 3.87, -2.37 |

**Table S6.** Single crystal data and structure refinement details for **MnL**_._

| Chemical formula | (C_42_H_65_N_9_O_28_Mn_3_) |
| --- | --- |
| *M*_r_ (g mol^-1^) | 1308.82 |
| Crystal system, space group | [Monoclinic](file:///C:\Users\Corey\Dropbox%20(Personal)\RS%20Open%20Science%20Paper%20June%202017\te1709c%20_space_group_crystal_system), *P*2*_1_*/*n* |
| Temperature (K) | 150 |
| *a, b, c* (Å) | 4.8941(4), 16.6396(13), 22.4311(15) |
| *β (°)* | 92.215(7) |
| *V* (Å^3^) | 1825.3(2) |
| Z | 4 |
| Radiation type | Mo *K*α |
| μ (mm^-1^) | 0.78 |
| Crystal size (mm) | 0.28 x 0.06 x 0.05 |
| Absorption correction | Gaussian |
| T_min_, T_max_ | 0.749, 1.000 |
| No. of observed measured, independent and observed [*I* > 2σ(*I*)] reflections | 16555, 4474, 2856 |
| *R*_int_ | 0.086 |
| (sinθ/λ)max (Å^-1^) | 0.696 |
| *R*[F2 > 2σ(F2)], ωR(F^2^), S | 0.083, 0.204, 1.09 |
| No. of reflections | 4474 |
| No. of parameters | 284 |
| H-atom treatment | H-atom parameters constrained |
| Δρ_max_, Δρ_min_ (e Å^-3^) | 0.87, -0.88 |

**Table S7.** Single crystal data and structure refinement details for **Cu_3_L_2_**_._

| Chemical formula | ([C_30_H_32_N_6_O_16_](file:///C:\Users\Corey\Dropbox%20(Personal)\RS%20Open%20Science%20Paper%20June%202017\adam_29jan15_a%20_chemical_formula_moiety)[Cu_3_](file:///C:\Users\Corey\Dropbox\RS%20Open%20Science%20Paper%20June%202017\adam_29jan15_a%20_chemical_formula_moiety)) |
| --- | --- |
| *M*_r_ (g mol^-1^) | 923.23 |
| Crystal system, space group | [Triclinic](file:///C:\Users\Corey\Dropbox%20(Personal)\RS%20Open%20Science%20Paper%20June%202017\te1709c%20_space_group_crystal_system), *P*-1 |
| Temperature (K) | 150 |
| *a, b, c* (Å) | 9.7937(11), 12.2893(14), 15.9285(17) |
| *α, β, γ (°)* | 100.636(7), 98.109(7), 107.225(6) |
| *V* (Å^3^) | 1760.1(3) |
| Z | 2 |
| Radiation type | Mo *K*α |
| μ (mm^-1^) | 1.88 |
| Crystal size (mm) | 0.1 x 0.08 x 0.06 |
| Absorption correction | Multi-scan |
| T_min_, T_max_ | 0.662, 0.746 |
| No. of observed measured, independent and observed [*I* > 2σ(*I*)] reflections | 29110, 7771, 5218 |
| *R*_int_ | 0.072 |
| (sinθ/λ)max (Å^-1^) | 0.643 |
| *R*[F2 > 2σ(F2)], ωR(F^2^), S | 0.046, 0.103, 1.03 |
| No. of reflections | 7771 |
| No. of parameters | 500 |
| H-atom treatment | H-atom parameters constrained |
| Δρ_max_, Δρ_min_ (e Å^-3^) | 0.84, -0.50 |

**Table S8.** Single crystal data and structure refinement details for **ZnL**.

| Chemical formula | ([C_14_H_15_N_3_O_6_Zn](file:///C:\Users\Corey\Dropbox%20(Personal)\RS%20Open%20Science%20Paper%20June%202017\tle1605c%20_chemical_formula_moiety)) |
| --- | --- |
| *M*_r_ (g mol^-1^) | 386.66 |
| Crystal system, space group | [Monoclinic](file:///C:\Users\Corey\Dropbox%20(Personal)\RS%20Open%20Science%20Paper%20June%202017\te1709c%20_space_group_crystal_system), *P*2*_1_* |
| Temperature (K) | 283 |
| *a, b, c* (Å) | 4.7261(7), 19.247(2), 8.7281(17) |
| *β (°)* | 104.839(17) |
| *V* (Å^3^) | 767.5(2) |
| Z | 2 |
| Radiation type | Mo *K*α |
| μ (mm^-1^) | 1.64 |
| Crystal size (mm) | 0.35 x 0.10 x 0.02 |
| Absorption correction | Gaussian |
| T_min_, T_max_ | 0.986, 0.999 |
| No. of observed measured, independent and observed [*I* > 2σ(*I*)] reflections | 4685, 2563, 1681 |
| *R*_int_ | 0.096 |
| (sinθ/λ)max (Å^-1^) | 0.589 |
| *R*[F2 > 2σ(F2)], ωR(F^2^), S | 0.092, 0.257, 1.000 |
| No. of reflections | 2563 |
| No. of parameters | 207 |
| H-atom treatment | H-atom parameters constrained |
| Δρ_max_, Δρ_min_ (e Å^-3^) | 092, -0.85 |
